# An Integrated Flow Cytometry-Based System for Real-Time, High Sensitivity Bacterial Detection and Identification

**DOI:** 10.1371/journal.pone.0094254

**Published:** 2014-04-09

**Authors:** Dan A. Buzatu, Ted J. Moskal, Anna J. Williams, Willie Mae Cooper, William B. Mattes, Jon G. Wilkes

**Affiliations:** 1 National Center for Toxicological Research, Food and Drug Administration (FDA), Jefferson, Arkansas, United States of America; 2 Vivione Biosciences, Pine Bluff, Arkansas, United States of America; 3 PharmPoint Consulting, Poolesville, Maryland, United States of America; Cornell University, United States of America

## Abstract

Foodborne illnesses occur in both industrialized and developing countries, and may be increasing due to rapidly evolving food production practices. Yet some primary tools used to assess food safety are decades, if not centuries, old. To improve the time to result for food safety assessment a sensitive flow cytometer based system to detect microbial contamination was developed. By eliminating background fluorescence and improving signal to noise the assays accurately measure bacterial load or specifically identify pathogens. These assays provide results in minutes or, if sensitivity to one cell in a complex matrix is required, after several hours enrichment. Conventional assessments of food safety require 48 to 56 hours. The assays described within are linear over 5 orders of magnitude with results identical to culture plates, and report live and dead microorganisms. This system offers a powerful approach to real-time assessment of food safety, useful for industry self-monitoring and regulatory inspection.

## Introduction

The technologies and practices used by the food industry have undergone dramatic changes in the last decade with regard to speed and production volume [1], yet with these changes product safety becomes an increasing concern [2]. According to the latest estimates from US Centers for Disease Control and Prevention (CDC), there are approximately 48 million cases of food poisonings per year which result in 128,000 hospitalizations and 3,000 fatalities [3]. Against this backdrop the US Congress passed the 2010 U.S. Food and Drug Administration (FDA) Food Safety Modernization Act, authorizing a combination of food producer performance standards, as well as FDA inspections designed to ensure those standards are met [4]. However, the law places significant technological demands on both the industry and the FDA [5]–[7].

Bacterial cell culture-based methods are considered the gold standard by the FDA and the US Department of Agriculture (USDA) for detection of pathogens in food; the FDA Bacteriological Analytical Manual (BAM) specifies procedures for *Escherichia coli* 0157: H7, *Salmonella enterica*, *Listeria monocytogenes*, *Campylobacter jejuni*, etc. [8]. The CDC uses similar methods to identify *Bacillus anthracis*, *Mycobacterium tuberculosis*, and other pathogens from clinical samples [9]. Bacterial culture identification is a lengthy process that includes multiple procedural steps. For example, the FDA BAM Chapter 4a requirement for diarrheagenic *E. coli* in food specifies culture plate confirmation after 48 to 56 hour even though it uses polymerase chain reaction (PCR) for screening presumptive positives and again for confirming culture plate positive isolates. This approach uses PCR to identifying specific pathogens via genetic elements markers, coupled with plate cultures to demonstrate the viability of those microbes. Although this approach produces accurate results and is authoritative for public health regulatory and enforcement purposes, its unacceptably long time to result (TTR) presents a burdensome impediment to timely food safety assessment.

Efforts have been made to reduce TTR while maintaining specificity and selectivity. Despite significant improvements, non-culture approaches developed to date have limitations that preclude real time, highly sensitive detection in food matrices. For example, immunochemical methods, such as enzyme-linked immunosorbent assays (ELISAs) and lateral flow immunochromatographic devices are able to display a rapid (∼15 min.) colorimetric response if the target pathogen is present [10] but in practice these techniques have poor sensitivity, require >10^5^ microorganisms total for identification, and are prone to cross-reactivity [11]. DNA testing such as PCR is used for definitive microorganism identification [12]–[14], yet contaminating DNA in the environment and laboratory can lead to false positive results, food components can disable PCR primers and lead to false negative results, and spontaneous mutations in genomic DNA can compromise detection [15], [16].

Many techniques are challenged by complex sample matrices and require preliminary extraction approaches that can add one or more days to TTR for selective targets [17], [18]. There are currently more than 20 Association of Official Analytical Chemists (AOAC) Performance Tested Methods commercially available for the detection of *E. coli* O157:H7 in one or more foods (e.g. ground beef, apple cider, orange juice, pasteurized milk, spinach, lettuce, and boneless beef trim). Most of these methods apply selective enrichment as a step to limit the growth of background microflora while permitting selective proliferation of the pathogens [19]–[21]. Such steps, while lengthening TTR, may still fail to detect low levels of viable pathogens which retain pathogenicity and can cause disease [22].

Flow cytometry, with its promise for single cell sensitivity with the possibility for detection with little or no enrichment or target isolation, has been used in attempts to detect microbial contamination[23]–[27]. However, real time detection of pathogens in complex matrices by flow cytometry has been hampered by a combination of instrumental performance limitations (particle size resolution, inter-instrumental equivalence); system ruggedness (optics stability to physical shocks and temperature variations, data acquisition complexity and operator competency), and confounding events from sample matrix particles. These challenges have thwarted development and deployment of a flow cytometry based system useful for early warning and quality assurance in food and clinical contexts.

Using a combination of fluorescent dyes sensitive to cellular state, polyclonal antibodies targeting specific pathogens, specific assay additives, and a carefully defined cytometer gating logic, we have developed a system for detecting target microbes in food production environments that: 1) requires no or minimal enrichment, 2) achieves single cell sensitivity, 3) delivers a quantitative response, 4) offers analytical specificity (i.e., target pathogens without false negative reports from non-target particles), 5) provides consistent and robust instrumental operation, 6) utilizes reasonably stable and relatively inexpensive reagents, and 7) does not require extensive operator training. Importantly, the complete analytical procedure for product testing delivers results in less than eight hours, suitable for real-time industrial applications. The novel sample preparation approaches, instrument operating parameters, and reagent qualities, termed RAPID-B, were developed under a National Center for Toxicological Research (NCTR)/FDA/HHS Cooperative Research and Development Agreement (CRADA) with Vivione Biosciences LLC, of Pine Bluff, AR, which is currently commercializing the technology. Two types of assays using this system demonstrate its performance and sensitivity.

## Methods

### Strains and Media

During the initial development of the pathogen specific assay, an avirulent strain of *E. coli* O157:H7 (ATCC 43888) was used. This strain is missing the Shiga toxin I and II genes and cannot produce Shiga-like toxin, so it was used to provide an additional layer of safety for assay development. The 43888 strain was also used for the growth curve experiment. For assay validation, a switch was made to ATCC 43895 which is an outbreak strain of *E. coli* O157:H7 that produces Shiga-like toxin I and II. The bacterial isolate used for the linearity chart was Arkansas Department of Health #3000372, EHEC *E. coli* O157:H7. The results presented for the TPC assay were *Ralstonia picketii*, a thermophile collected from card stock produced by a local paper mill.

No permissions were necessary to obtain the cardstock as the paper company was participating in a study with our lab and the card stock was delivered directly to the lab. Brain Heart Infusion (BHI) broth was used as the growth medium for *E. coli* O157 with incubation at 42°C to promote fast growth in the development and validation of the assay. TSB and trypticase soy agar (TSA) were used for the growth curve experiments at 42°C. PCA and SMAC were used for the linearity study at 37°C.

### Flow Cytometry

**Figure 1:**
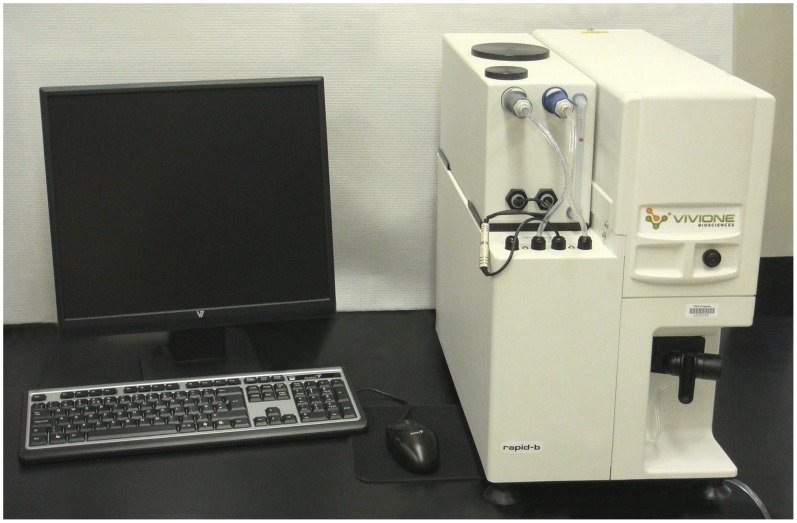
Photograph of the Vivione Biosciences 9013 Flow Cytometer.

There are several flow cytometric performance features that are required for bacterial pathogen detection: 1) sub-micron particle resolution, 2) a large flow cell configuration that does not clog, and 3) high speed sample introduction and detection, concepts essentially first described by Steen and Lindmo [27]. The RAPID-B model 9013 flow cytometer (Vivione Biosciences, LLC, Pine Bluff, AR) was chosen for meeting these performance requirements with oil immersion optics and jet nozzle design in addition to other features. The optics and fluidics of the instrument are attached vertically to a thick metal support that provides physical and thermal stability. Recalibration is not necessary and the instrument can be transported in a vehicle to a field location and used. A photograph of this instrument is shown in **Figure 1**.

**Figure 2:**
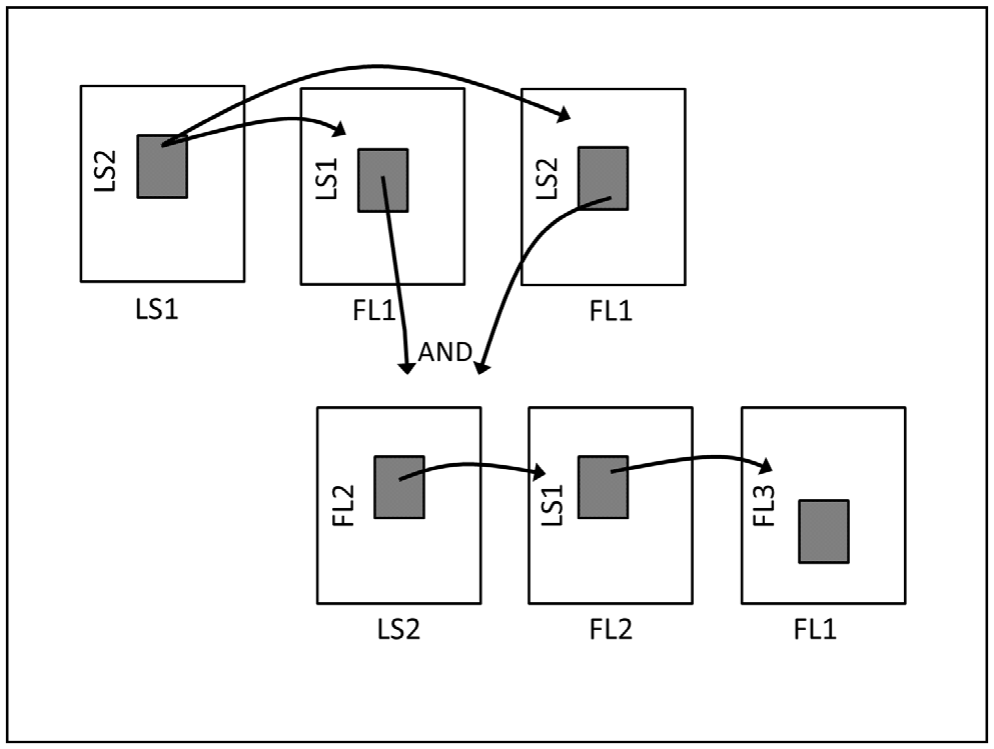
Flow cytometer gate logic for RAPID-B assays. The flow of information is indicated by the arrows. LS1 and LS2 represent standard flow cytometry scatter channels side and forward scatter, respectively. FL1, FL2, and FL3 represent standard green, yellow, and red fluorescence flow cytometry channels, respectively.

**Figure 3:**
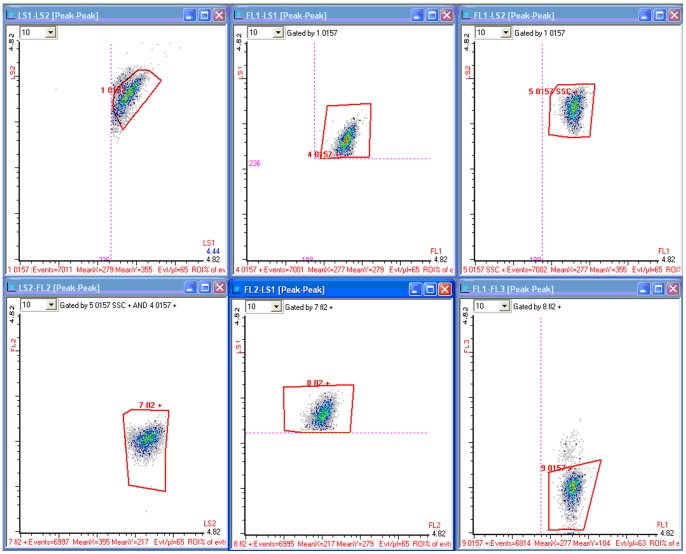
Screen capture from the 9013 flow cytometer showing the flow of information. The sample is *E. coli* O157. Information starts at the scatter plot (upper left) and then passes through gates that are a combination of scatter and fluorescence regions to the final target region in the fluorescence plot at the bottom right. The count of events progresses from 7011 in the left upper panel to 6814 in the final counting region. The order of the panels corresponds to the order of gating logic in Figure 2.

As with many conventional cytometry assays, development of the serial gate logic shown in **Figure 2** started with plotting the side scatter (LS1 on the 9013) versus forward scatter (LS2 on the 9013) signatures of the sample. Using thoroughly washed, highly concentrated isolates of target bacteria, LS1 and LS2 photo multiplier (PMT) voltages were adjusted to bring the scatter population to the center of the plot. Once these voltages were adjusted, a region of interest was drawn around this concentrated population with borders slightly wider than the population. For example, the region of interest can be expanded for targets like *Salmonella* spp. so inclusivity is retained for all applicable strains. This was done using multiple population location experiments for a representative set of target cell isolates. This information was then passed onto two separate plots representing LS1 vs. FL1 and LS2 vs. FL1 (the 9013 green fluorescence color channel centered around 525 nm). Boxes were drawn around these populations in their respective plots. The information from each LS1 vs. FL1 and LS2 vs. FL1 gate was then combined using an AND Boolean function on another plot representing LS2 vs. FL2 (yellow fluorescence color channel ∼600 nm). A box was drawn around the population on the LS2 vs. FL2 plot and information from this plot was passed to another plot representing LS1 versus FL2. A region of interest was drawn around this population and the information was then passed to a final plot representing FL1 vs. FL3 (red fluorescence color channel >670 nm). The target population had a region of interest drawn around it that is slightly larger than a concentrated sample. **Figure 3** provides a series of plots from the electronic gates of the RAPID-B *E. coli* O157 assay. It shows the changing appearance of concentrated sample, events transmitted through the series of gates as described above.

**Figure 4:**
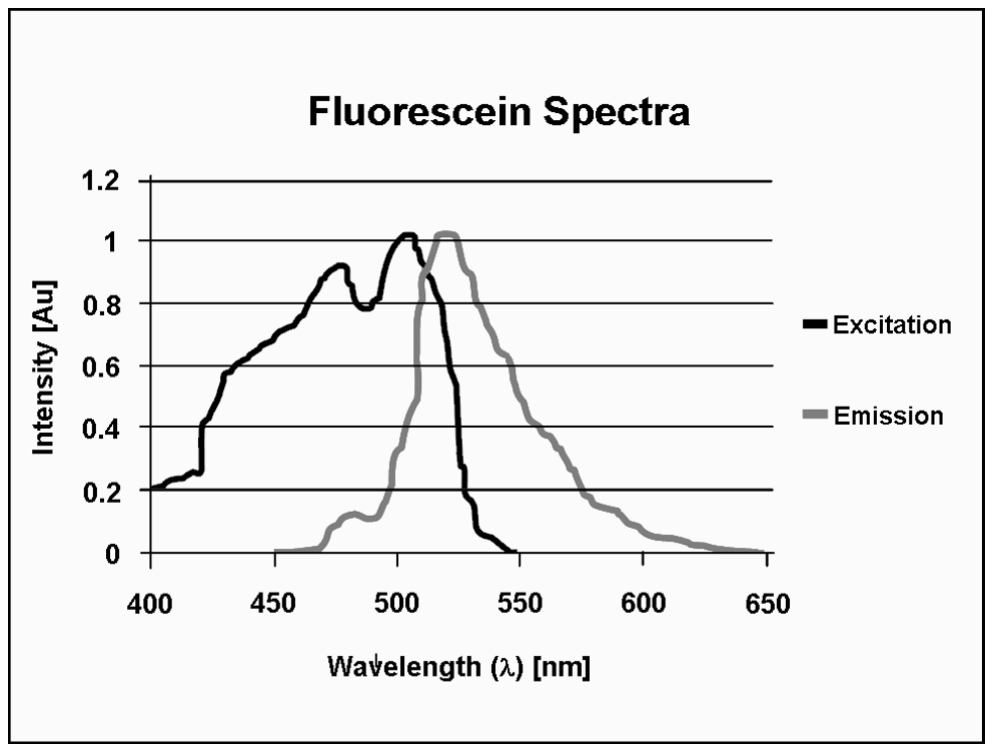
Fluorescent excitation and emission spectra of fluorescein isothiocyanate.

The logic behind this series of gates relies on the fact that a fluorescence probe such as fluorescein isothiocyanate (FITC) (e.g., the RAPID-B *E. coli* O157 assay) most strongly emits around 525 nm, but the emission peak **Figure 4** is somewhat broad and trails off gently from 525 to 600 nm. Reliable detection of FITC labeled pathogens interrogates the strength of both FL1 and FL2 fluorescence. Passing the scatter and fluorescence information previously described through the series of gates helps to dramatically reduce particulate noise that is evident in individual color channels. This complexity is required by the need to exclude very high percentage (99.999%) of coincident matrix events.

### Total Plate Count Assay

The bacterial total plate count (TPC) reagent was created by combining two DNA dyes (thiazole orange (TO), and propidium iodide (PI)), Tween 20 (to marginally increase bacterial cell permeability), and EDTA (for cell surface conditioning). TO enters live bacteria, binds to DNA and fluoresces green (fluorescence channel 1, FL1, 525 nm). If the bacterium is stressed or dead, its cell wall degrades and PI also enters the cell, intercalates with DNA, quenches the TO signal, and emits a red signal (fluorescence channel 3, FL3, >670 nm). The combined approach functions as a live/dead cell count assay. TO and PI and other DNA dyes are used by other manufacturers to produce cell viability kits [28]. This strategy is not species-specific but generally useful for enumerating vegetative cells as the dyes will not label spores efficiently. To obtain reliable live, injured, and dead cell counts in diverse biological matrices, this assay method relies on not only the gating logic described but also novel sample cleanup approaches.

Suspect contaminated samples were suspended in 1 ml of PBS and placed in a 2 ml Eppendorf microcentrifuge tube. Concentration of target cells from larger volumes of liquid can be accomplished using centrifugation. Bacterial cell pellets were dispersed by intense vortexing or pipette trituration. After dispersion, 330 μl of TPC reagent were added, the mixture was vortexed gently for 5 min, removed from the vortexer, and analyzed with the RAPID-B instrument using a protocol specific for this assay. Total time to result per sample is 7 min (2 min for analysis and 5 min for incubation). When running multiple samples, staggered incubation times are recommended to take advantage of the quick analysis time on the instrument. Using a staggered incubation procedure, 15 to 20 samples per hour can be analyzed.

### Pathogen-Specific Assay

Protocols were developed to produce accurate pathogen-specific assays with no false positive or false negative results. Several parameters were optimized including: 1) comprehensive titration of antibody concentrations, 2) antibody incubation times, decreased from half an hour or more to only a few min to increase assay selectivity by reducing the opportunity for cross-reactivity, and 3) optimized gentle agitation during reagent-analyte incubation.

The pathogen specific (PS) assay uses a procedure similar to that of the TPC assay. Samples are suspended in 1 ml PBS, followed by 10 μl of PS reagent A and 240 μl of reagent B. PS reagent A is a combination of dilute polyclonal antibodies and bovine serum albumin. PS reagent B is a combination of surfactants, PI and EDTA. The sample tube is vortexed gently for 6 min and then analyzed on the RAPID-B instrument in a manner similar to that of the TPC assay, but using a PS-specific protocol (e.g. *E. coli* O157 protocol). Total time to result was 8 min (2 min instrumental analysis +6 min incubation).

### Direct Detection

Surfaces were wiped with a filter swab, then returned to the sample holder with filter containing PBS and agitated to release cells. The holder was inverted to push the liquid through a 5 micron filter base directly into a microcentrifuge tube containing reagents, vortexed for 5 min, and analyzed directly on the instrument.

### Pulsification

A combined mechanical/chemical technique was developed to extract bacteria from foods. A Pulsifier (Microgen Bioproducts Ltd, Camberley Surrey, UK) can be used to effectively release bacteria from food. The oscillating metal ring of the Pulsifier induces high frequency shock waves through the sample bag containing food, releasing bacteria with minimal perturbation of the food matrix. Addition of a surfactant such as Tween 20 to the sample bag facilitates the extraction of virtually all cells from the food matrix. For example 10 ml of Tween 20 at 0.1% concentration in water can be added to the bag containing 25 grams of food product in 75 ml of BHI prior to pulsification.

### Phloxine Photobleaching

The use of Phloxine B in reducing background from colored food materials has been described [29]; briefly, following pulsification, 42.5 ml of broth (approximately half the sample volume) was poured out of the Whirlpak bag containing 25 grams of spinach into a 50 ml conical centrifuge tube. To this tube 12.5 μl of 0.01% Phloxine B was added, and the tube was placed into an illumination chamber. This black metal chamber has two compartments. The top compartment contains a 250 W halogen light facing down, separated from the sample chamber by a ¼ inch glass plate to keep heat away from samples. Additionally, the light chamber and sample chamber are each vented using fans in the side walls to prevent the heating of samples during illumination. The sample was illuminated with bright light (4000 lumen) at a distance of 10 cm for 1 min and then transferred to the next step of the procedure.

### Percoll Gradient Centrifugation

Following the phloxine photobleaching, samples were filtered and layered on top of a Percoll (GE Healthcare Biosciences, Pittsburgh, PA) layer, then centrifuged at 16,100 g for 1 min (see Lindqvist [30]). Bacteria were effectively separated through the Percoll layer while the interfering food matrix components remained on top.

**Figure 5:**
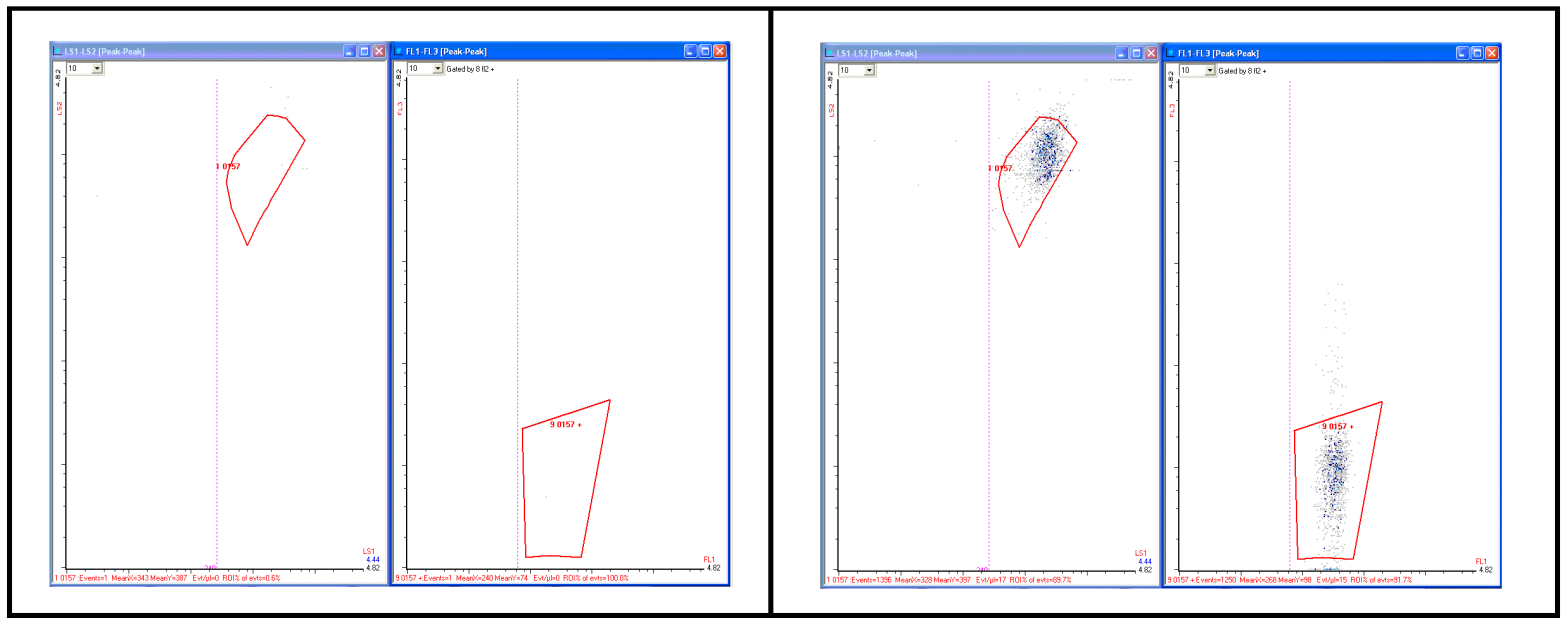
Effects of background reduction. Forward versus side scatter plots (left panels) and Fluorescence (FL3 vs. FL1) intensity plots (right panels) of the RAPID-B Pathogen Specific (PS) assay for *E. coli* O157:H7 stx1+ and stx2+ (ATCC 43895). A. Sterile spinach sample blank (25 g) B. Spinach sample (25 g) spiked with trace (single digits) of the *E. coli* O157 ATCC 43888 strain. Both samples were incubated in 75 ml BHI for 6 hr, subjected to phloxine photobleaching, followed by Percoll gradient centrifugation.

The combined result of phloxine treatment and Percoll centrifugation on the pathogen specific assay can be seen in **Figure 5** **(A and B**).

## Results

### Improvements of RAPID-B Compared to Standard Protocols

**Figure 6:**
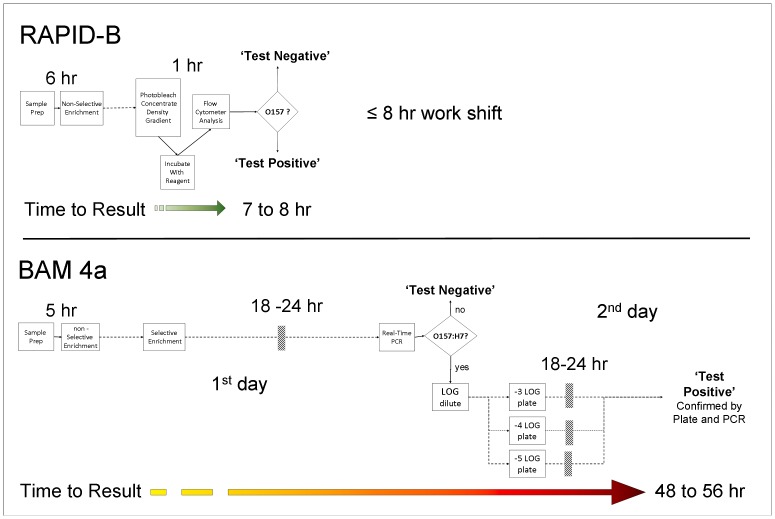
Time to results (TTR) for both RAPID-B and FDA BAM pathogen contamination assays. The sequence of steps in the RAPID-B Total Plate Count Assay and Pathogen-Specific Assay are contrasted to those in the FDA BAM 4a assay.

The overall flow of operations in the RAPID-B system, for both the Total Plate Count Assay, as well as the Pathogen-Specific Assay is outlined in **Figure 6** with a comparison to FDA’s BAM Chapter 4A (Diarrheagenic *Escherichia coli*).

Since, many foods and biological matrices (sputum, for example) contain particulate components similar in size and fluorescence to labeled bacterial cells, a serial gate logic was required to reduce the likelihood that such components would meet the target signal criteria. The novel serial gate logic that was developed effectively isolates signals for the target microbe by reducing particulate noise (see Methods and **Figure 2**) and can be applied to different sample workups as described below. The result is a fast flow cytometric assay capable of detecting single cells and providing results without false positive or negative interference.

### Total Plate Count Assay

**Figure 7:**
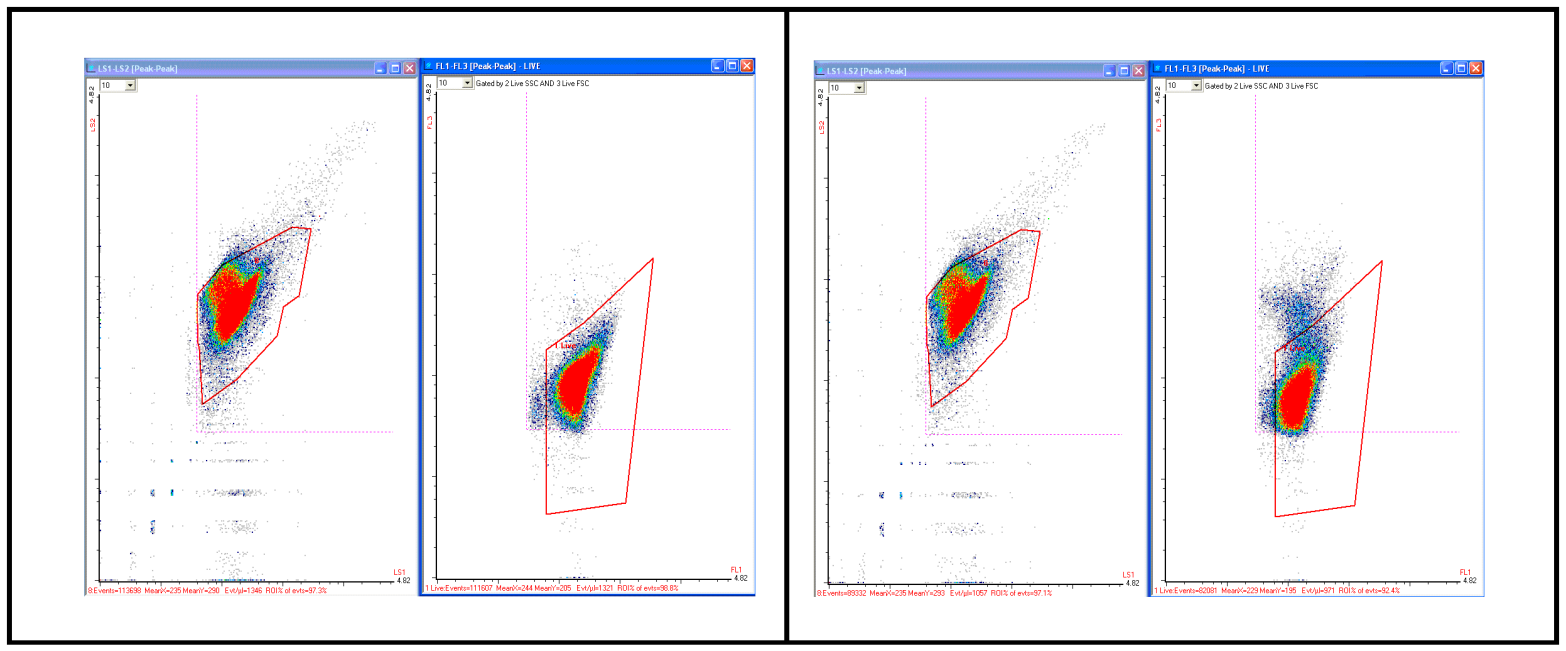
Representative flow cytometry data for RAPID-B Total Plate Count assay. Scatter (left panels) and fluorescence intensity (right panels) plots of the RAPID-B Total Plate Count (TPC) assay for *Ralstonia picketti*. **A.** Non-stressed *Ralstonia* sample (111,607 live, 558 injured (0.5%)). **B.** Heat stressed *Ralstonia* sample, events above the counting region due to PI penetrating damaged cell walls (82,081 live, 6894 injured (8.4%)).

**Figure 7** **(A and B)** depicts two representative dot plots of a typical TPC assay, the left hand plot in each case indicating intensity from two light scatter sensors and the right hand, coincident signals in two fluorescence sensors (FL1 vs. FL3) enumerating viable and dead cells from a paper carton sample manufactured by a local paper mill. The predominant bacteria present was determined by the NCTR Division of Microbiology using conventional techniques to be *Ralstonia picketii*, aerobic proteobacteria commonly found in paper mill effluents [31]. This bacteria metabolized organic compounds found in the paper fiber, continued to reproduce at room temperature when the card stock was immersed in distilled water, and proved heat resistant; colonies could be maintained at 50°C [31]. **Figure 7A** shows results for a TPC assay for a 1 ml sample from a 2 day culture of *Ralstonia* (card stock immersed in 100 ml of water); **Figure 7B** represents results for a 1 ml sample culture but heat treated at 60°C for 30 min. Dots associated with heat stressed injured and dying bacteria rise in a plume above the live target counting region in **Figure 7B**. Comparison of the plots quantifies in real time the efficacy (or lack thereof) of heat based disinfection for this thermophile. Not all the cells were killed, even by treatment at 95°C for 30 min (data not shown).

### Pathogen-Specific Assay

The development of pathogen-specific detection assays required the use of antibodies specific to the target pathogens (e.g. *Listeria monocytogenes*, *E. coli* O157, *Salmonella* spp.) Polyclonal antibodies are used due to their enhanced avidity for cell surface epitope binding (KD≈10^−9^).

**Figure 8:**
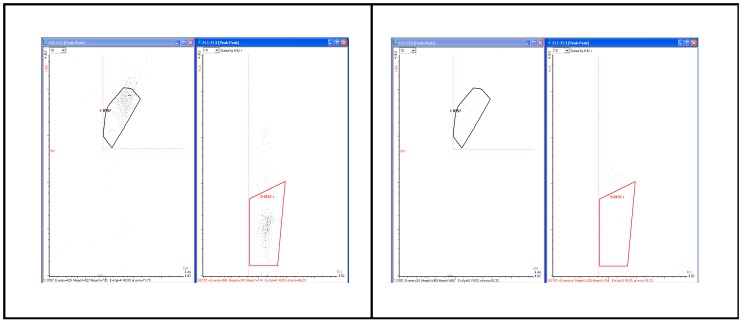
Representative flow cytometry data for RAPID-B Pathogen Specific assay. Scatter (left panels) and fluorescence intensity (right panels) plots of the RAPID-B Pathogen Specific (PS) assay for *E. coli* O157:H7 stx1+ and stx2+ (ATCC 43895); results from a spinach validation study. **A.** Positive spinach sample (368 live *E. coli* O157 cells, 48 dead). **B.** Negative spinach sample (4 events; a threshold of 6 events was used for blanks based on historical data).

**Figure 8** **(A and B)** depicts two representative dot plots of a typical PS assay, the left hand plot in each case indicating intensity from the light scatter sensors and the right hand, coincident signals in two fluorescence sensors (FL1 vs. FL3) enumerating viable in the trapezoidal counting region and dead cells above the target region. Each dot in this counting region represents a live *E. coli* O157 cell, and each dot above the counting region represents a dead or injured cell. As can be seen, contamination was detected in the 8 hour assay with a low background.

**Figure 9:**
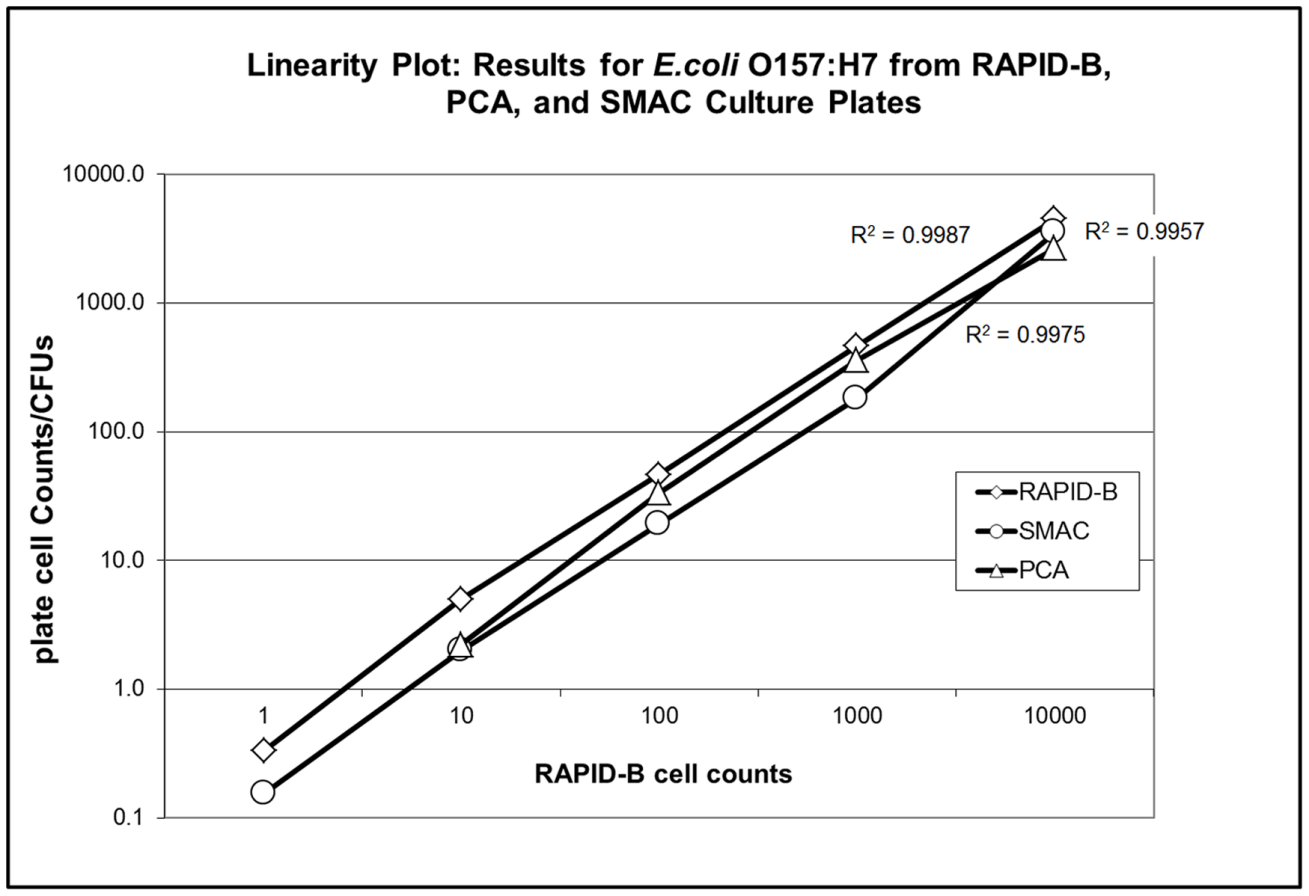
Linearity of the RAPID-B *E.coli* O157 assay. The linearity of the RAPID-B E.coli O157 assay is demonstrated and compared with 2 different types of culture plates. The bacterial isolate used was Arkansas Department of Health #3000372, EHEC E.coli O157:H7. Each data point for the plate count agar (PCA) and Sorbitol-MacConkey Agar (SMAC) is an average of 6 plate counts.

Linearity of the RAPID-B *E. coli* O157 assay was assessed as part of a larger USDA Food Emergency Response Network (FERN) level 2 validation performed by the Arkansas Department of Health (ADH). A report of this validation is available online as supplemental material accompanying a manuscript by Wilkes et al. [32]. **Figure 9** shows the linearity of the RAPID-B *E.coli* O157 assay over five orders of magnitude in comparison to plate-based assays. A total of 649 RAPID assays and 267 culture plates were used with three test panels and testing performed on three different dates. RAPID-B tests were performed on two instruments both giving consistent results. RAPID-B results are parallel to the plate count agar (PCA) and Sorbitol-MacConkey Agar (SMAC) results have a magnitude in cell count greater than plate counts by 1.1 to 1.5 times. This indicates 10 to 50 percent greater counts from the RAPID-B assay compared to either PCA or SMAC culture plate arrays. The decreased but parallel plate count is most likely due to the superior growth characteristic in liquid media compared to that with plate culture media [33], [34].

**Figure 10:**
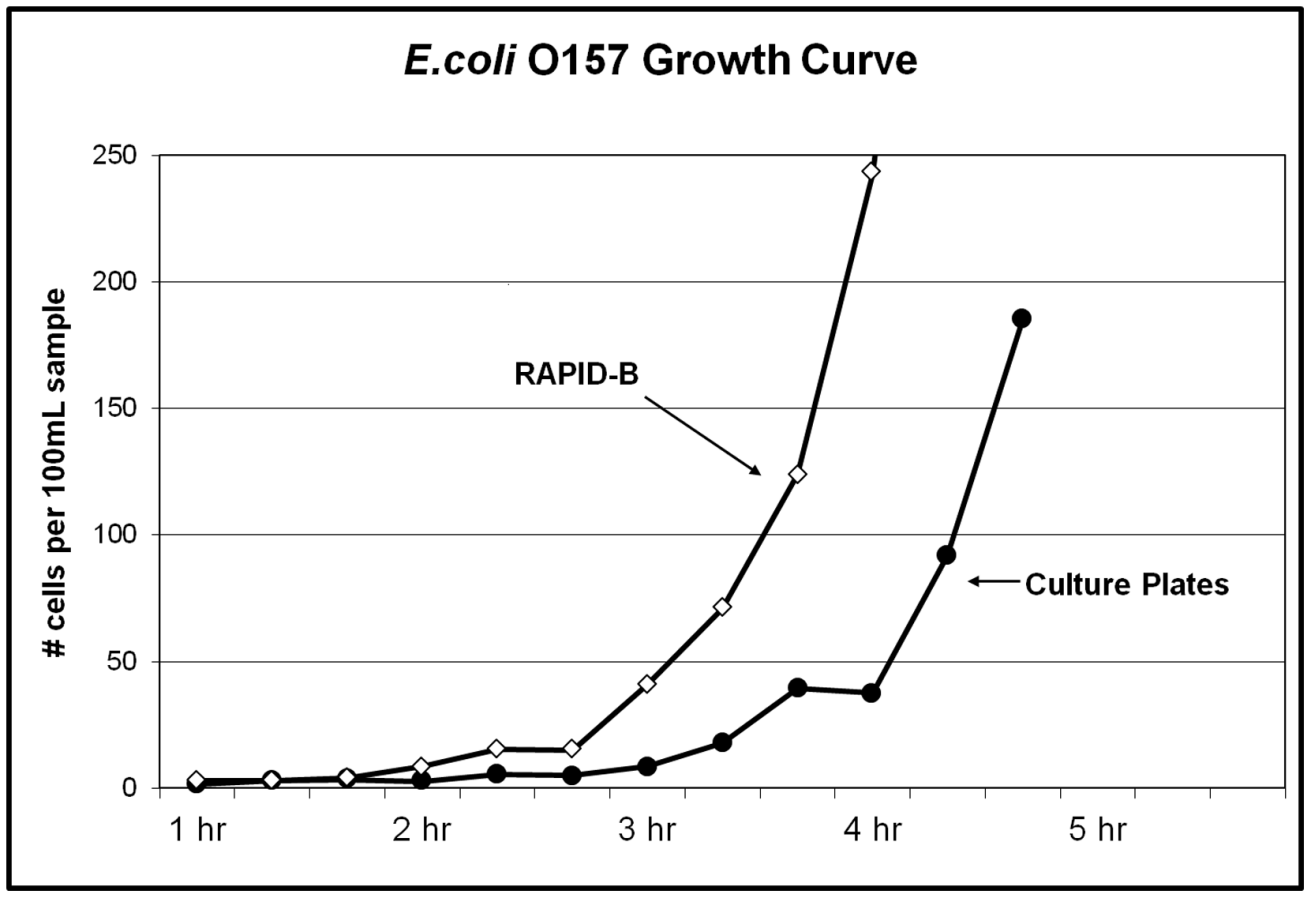
Growth curve of *E*.*coli* O157. As measured by RAPID-B (real time) and culture plates (historical). Two RAPID-B measurements were averaged at each time point for the RAPID-B curve, and two PCA culture plates are averaged for the culture plate curve.

An experiment was performed to assess the lag phase associated with *E. coli* O157 growth in real time. Due to the sensitivity of the system, dilutions of ATTC 43888 strain (*E. coli* O157 attenuated to remove the shiga-toxin producing gene) were titrated down to less than 10 cells in 100 μl (the sample volume that runs through the system in 1 min). All dilutions were confirmed by PCA culture plates; the experiment started with an average 3 cells per 100 μl. **Figure 10** is a plot representing the course of this experiment with measurements taken every 20 min for a total time over 3 hours.

Over the range displayed in **Figure 10**, the RAPID-B results average 29.5% relative standard deviation (RSD), and PCA results average a similar 30.9% RSD. It is important to point out that *E. coli* O157 ATCC 43888 cells are normally grown and maintained in our laboratory in BHI, a preferred growth medium. In order to stress the cells and replicate real world conditions such as food packaging, storage, and refrigeration, dilute concentrations of the cells were refrigerated 24 hour prior to the start of the experiment. In order to initialize the start of growth, the growth medium was switched from BHI to tryptic soy broth (TSB). The broth was pre-warmed to 42°C, providing faster growth for *E. coli* O157. The sudden exponential growth associated with the end of the lag phase appears to occur somewhere between 2 hour and 2 hour 20 min as measured with the RAPID-B approach and between 3 hour 20 min and 3 hour 40 min as measured with culture plates. There are several factors that may have played a role in the longer lag phase associated with the plate growth. These include quorum sensing, surface tension, exposure to nutrients (total immersion vs. partial exposure on plates) oxygen exposure levels, etc. Subsequent experiments using dilute concentrations of cells grown in liquid broth for 6 hours have confirmed the lag phase ends just after 2 hr. Further experiments performed to detect 1 cfu of *E. coli* O157:H7 in 25 grams of food product confirmed a 6 hour outgrowth period as sufficient to detect contamination, contingent on a zero or single digit fluorescent background from the food or biological matrix.

### Direct Detection

In situations where microbial loads are higher than trace level and there are no matrix interference issues, the RAPID-B system can be used to perform direct detection of pathogens in minutes, including food products, environmental testing and food contact surfaces, clinical samples, etc. Samples can be obtained with swabs or sponges from any other food product or contact surface that does not require cleanup or enrichment to facilitate analysis. Processing involves rapid filtration and mixing with reagents, all accomplished in minutes, and is followed by direct analysis on the flow cytometer (data not shown).

### Food Product Analysis with Background Reduction

In keeping with one of the FDA’s primary missions, the RAPID-B system was developed to detect pathogens in a food production context. Food and biological samples introduce a number of challenges to flow cytometers such as particulate matter that can clog sample lines and flow cells, and particles similar in size and intrinsic fluorescence to labeled bacteria. If not eliminated, these particles effectively mimic labeled bacteria and can lead to false positive results. Conversely, unrecoverable bacteria trapped in a food matrix can lead to false negative results. Extraction and sample cleanup methods are required to enable fast detection in these matrices with no false positive or negative results.

Pulsification for 1 min resulted in minimal disruption of the sample matrix (either food or biological) while efficiently extracting cells; however, this step left a significant amount of matrix particles dispersed into the sample liquid. The background fluorescence associated with these particles needed to be reduced or eliminated to allow meaningful detection and enumeration of target bacteria. A two-step procedure was developed to reduce background fluorescence, incorporating a photobleaching step followed by density gradient separation. Phloxine B, a singlet oxygen producing photoreactive dye (λmax 560 nm; quantum yield 0.59) approved for use in consumer products was found to be effective in reducing background fluorescence without affecting bacterial counts [29]. For example, in low concentrations (e.g. 0.01% by weight), phloxine B phototreatment for 1 min changed the fluorescent emissions of particles released from chlorophyll-containing green leafy vegetables from green (conflicting with the pathogen detection color channel FL1) to deep red which is high on the FL3 fluorescence channel and beyond pathogen detection range. The impact of phloxine B phototreatment on reducing background fluorescence from spinach can be seen in **Table 1**. Phloxine B phototreatment was also effective in eliminating the background autofluorescence inherent in beta-carotene containing foods such as carrots (data not shown).

**Table 1:** Flow cytometer events for a negative control sample carried through the pathogen specific protocol.

Data Processing Cleanup Procedure	Scatter (events)	Fluorescence (events)
No electronic filters	148,071	5,936
With electronic filters	148,066	2,761
Photobleaching	2,843	35
Gradient Centrifugation	1	1

The numbers of events in the scatter and fluorescence regions are given for a negative control spinach sample processed through the pathogen specific protocol; note the reduction in both particulate matter and fluorescence interference as the sample moves through each of the steps.

While photobleaching dramatically reduced interfering fluorescence from the food matrix, a significant number (usually double digits) of non-bacterial particles that remained interfered with the final counting regions of these assays making trace level detection of pathogens problematic. Adding a Percoll density gradient centrifugation step (adapted from that of Lindqvist [30]) following phloxine photobleaching reduced both scatter and fluorescence counts to virtually zero (**Table 1**).

## Discussion

According to the latest estimates from CDC, 1 in 6 people (48 million) get food poisoning each year. The majority of all illnesses are caused by 31 pathogens, including viruses, bacteria, and parasites, with Norovirus and *Salmonella* spp. among the top offenders. While traditional microbiological methods have worked well for decades to detect and stop pathogen contamination in food, the speed of modern food production practices requires dramatic improvements in speed and accuracy of microbiological methods to assure the same safety standards. The NCTR Innovative Safety and Technologies Branch explored several highly-sensitive laboratory techniques including mass spectrometry and flow-cytometry methods to meet the standards for real time analysis results, high-throughput rates, and targeted unmatched accuracy for zero error rates in detecting pathogen outbreaks.

RAPID-B combines flow cytometry, enhanced detection techniques and background reduction that functions accurately to provide results that are as precise as culture plates but that require substantially less time. Results show how RAPID-B analysis can be performed directly in a complex food matrix environment in 6 hour with the ability to detect one bacterial cell. The system is portable and sufficiently rugged that it does not necessarily require the work to be performed in a food safety laboratory nor does it require highly-skilled personnel. The system consists of reagent test kits comprised of DNA dyes, antibodies, other proteins and specialty chemicals as already described in the assay sections. It also consists of the instrument, software components and instrument data collection protocols. Currently, RAPID-B assays are commercially available for the *E. coli* O157 Pathogen-Specific Assay and the Total Plate Count Assay. In addition prototype assays for other foodborne pathogens including *Listeria* spp, *Salmonella* spp., generic *E. coli*, *Campylobacter* spp., *Vibrio* spp., *Vibrio cholerae*, *Shigella* spp. and *Staphylococcus aureus* are under development. As different bacterial pathogens emerge as specific problems, appropriate RAPID-B tests can be developed. The compelling value of RAPID-B for food safety and clinical applications is the greatly reduced time to results. Real time results facilitate greatly improved process and product control for food manufacturers and dramatically higher through-put and accurate screens for regulatory authorities.
